# The Guiding Symptoms of Our Materia Medica

**Published:** 1881-11

**Authors:** 


THE GUIDING SYMPTOMS of our MATERIA MEDICA. By C. Hering. Vol. III. Philadelphia. The American Homœopathic Publishing Society.

This, the third volume of the "Guiding Symptoms," has recently appeared, after much delay. Let us hope that the other volumes will be more speedy in coming forth. The homœopathic profession should subscribe for this work; it is needed by all. And they should demand that the work be given them as Dr. Hering left it. The editors say in their preface: "Actuated, as we have been, by the spirit of the departed author, it has been our aim to complete the volume just as it would have been had Hering lived."

This volume carries the work from *Bryonia* to *Chamomilla,* inclusive. We hope, now that a third volume is out, that there will be a large increase of subscribers to the work. Send your orders to J. M. Stoddart & Co., 727 Chestnut street, Philadelphia.

Dr. Hering wrote, in preface to the first volume: "This work will especially commend itself to the busy practitioner, because it is an attempt to give our materia medica in such a form as will make the selection of the curative medicine in any given case as easy as possible. It is a *complement* to all other works on our materia medica, being principally a collection of cured symptoms. A symptom only cured has never such intrinsic value as *one produced and* cured, and yet, such a one must not be ignored." Clinical symptoms are useful in aiding us to use proved drugs. No drug has yet been exhaustively proven. Clinical symptoms are to the therapeutist as crutches to the lame, an assistance ; let us beware lest we rely so much on our " crutches" that we neglect to seek sound limbs. Those who advocate the use of unproved drugs, relying upon clinical symptoms solely, may be said to prefer " crutches" to sound limbs.

